# Bioaccumulation of heavy metals and ecophysiological responses to heavy metal stress in selected populations of *Vaccinium myrtillus* L. and *Vaccinium vitis-idaea* L

**DOI:** 10.1007/s10646-017-1825-0

**Published:** 2017-06-17

**Authors:** Marta Kandziora-Ciupa, Aleksandra Nadgórska-Socha, Gabriela Barczyk, Ryszard Ciepał

**Affiliations:** Department of Ecology, University of Silesia, Bankowa 9, PL 40-007 Katowice, Poland

**Keywords:** Heavy metal, Antioxidant response, *Vaccinium myrtillus*, *Vaccinium vitis-idaea*

## Abstract

The aim of this study was to determine the concentrations of heavy metals (Cd, Pb, Zn, Fe, and Mn) in soil, and their bioavailability and bioaccumulation in *Vaccinium myrtillus* L. and *Vaccinium vitis-idaea* L. organs. Analysis also concerned the physiological responses of these plants from three polluted sites (immediate vicinity of a zinc smelter in Miasteczko Śląskie, ArcelorMittal Poland S.A. iron smelter in Dąbrowa Górnicza-Łosień, and Jaworzno III power plant in Jaworzno) and one pseudo-control site (Pazurek nature reserve in Jaroszowiec Olkuski). All of the sites are situated in the southern parts of Poland in the Śląskie or Małopolskie provinces. The contents of proline, non-protein thiols, glutathione, ascorbic acid, and the activity of superoxide dismutase and guaiacol peroxidase in the leaves of *Vaccinium myrtillus* L. and *Vaccinium vitis-idaea* L. were measured. In soil, the highest levels of Cd, Pb, and Zn (HNO_3_ extracted and CaCl_2_ extracted) were detected at the Miasteczko Śląskie site. At all sites a several times lower concentration of the examined metals was determined in the fraction of soil extracted with CaCl_2_. Much higher Cd, Pb, Zn and Fe concentrations were found in *V. myrtillus* and *V. vitis-idaea* grown at the most polluted site (located near the zinc smelter) in comparison with cleaner areas; definitely higher bioaccumulation of these metals was found in lingonberry organs. Additionally, we observed a large capability of bilberry to accumulate Mn. Antioxidant response to heavy metal stress also differed between *V. myrtillus* and *V. vitis-idaea*. In *V. myrtillus* we found a positive correlation between the level of non-protein thiols and Cd and Zn concentrations, and also between proline and these metals. In *V. vitis-idaea* leaves an upward trend in ascorbic acid content and superoxide dismutase activity accompanied an increase in Cd, Pb, and Zn concentrations. At the same time, the increased levels of all tested metals in the leaves of *V. vitis-idaea* were accompanied by a decreased activity of guaiacol peroxidase. In both species increased Mn accumulation caused a decrease in antioxidant response.

## Introduction

*Vaccinium myrtillus* L. (bilberry) and *Vaccinium vitis-idaea* L. (lingonberry) are the most frequent and abundant dwarf shrubs species in the understory of conifer forests in Europe and Northern Asia, both belonging to the family Ericaceae and the genus *Vaccinium* (Liu et al. 2014; Rodriguez and Kouki 2015). Bilberry and lingonberry prefer silicate, nutrient-poor and humid soils. Although these species often co-occur, *V. myrtillus* generally requires moister conditions and shady sites, while *V. vitis-idaea* can also be found on more arid and exposed sites (Sebald et al. 1993; Polatschek 1999; Ganthaler and Mayr 2015). Dwarf shrubs provide provisioning (food), regulating (pollination), species habitat and they constitute one of the major drivers in the ecosystem dynamics of the boreal forest, affecting seedlings regeneration and the soil nutrient and carbon cycles (Nilsson and Wardle 2005; Kolari et al. 2006; Rodriguez and Kouki 2015). The major reason for the interest in *V. myrtillus* L. and *V. vitis-idaea* L. is that they can serve as model species of the forest floor of boreal forests, differing in their life strategies (Taulavuori et al. 2013). Bilberry is a deciduous species as it sheds its leaves during autumnal preparation for winter, whereas lingonberry, an evergreen species, retains its leaves for many years (Taulavuori et al. 2013; Parzych 2014). Because of these differences it is interesting to study whether or not these species vary in responses to any stress, including heavy metals stress.

Heavy metal pollution is one of the most important environmental problem these days. It is growing with inevitable pace, endangering humans, animals, and plants (Anjum et al. 2015a, b, 2016). The biological effects of individual metals are more or less known, but even though combinations of heavy metals are common in nature, their combined effects still need to be thoroughly investigated (Wilde et al. 2006; Cyjetko et al. 2010; Tkalec et al. 2014). All non-essential heavy metals, as well as essential ones, when present in higher concentrations than optimal, affect different cellular components, thereby interfering with the normal metabolic functions of plant cells (Tkalec et al. 2014). For example, elevated levels of heavy metals are associated with the increased generation of reactive oxygen species (ROS) (Emamverdian et al. 2015). These ROS interfere with various macromolecules and disrupt normal cellular functions and metabolism (e.g. resulting in lipid peroxidation, inactivation or damage of proteins and chlorophyll, DNA injury) (Anjum et al. 2016). Once formed, ROS must be detoxified as efficiently as possible to minimize damage. Antioxidant systems in plants are complex and involve an array of non-enzymatic and enzymatic mechanisms capable of preventing the cascades of uncontrolled oxidation (Gratão et al. 2005, 2008; Kandziora-Ciupa et al. 2013). The non-enzymatic antioxidants are generally small molecules that include tripeptide glutathione, proline, cysteine, ascorbate, non-protein compound rich in –SH groups, etc. The enzymatic antioxidant components contain the enzymes capable of removing, neutralizing, or scavenging ROS and the intermediates such as superoxide dismutase (SOD), catalase (CAT), guaicaol peroxidase (GPX), and glutathione reductase (Das et al. 2015). Antioxidant systems in plants may be used as early indicators of environmental stress on target organisms preceding morphological or ultrastructural damage, and as warning indicators for the ecosystem (Białońska et al. 2007; Kandziora-Ciupa et al. 2016). Estimation of plant ecophysiological responses in a field study may be useful in pollution biomonitoring as well as in verifying the effect of metal contamination on plant physiology (Hu et al. 2014; Nadgórska-Socha et al. 2016).

Therefore, the objective of the current study was to determine metal accumulation efficiency in polluted and non-polluted areas and ascertain the influence of selected heavy metals (Cd, Pb, Zn, and Mn) on levels of antioxidants (proline, non-protein thiols, glutathione, ascorbic acid) and antioxidant enzymes activity (guaiacol peroxidase, superoxide dismutase) in *Vaccinium myrtillus* L. and *Vaccinium vitis-idaea* L. leaves. We hypothesized that (1) bioaccumulation of chosen heavy metals in the leaves, stems, and roots of *Vaccinium myrtillus* L. and V*accinium vitis-idaea L*. from the polluted sites would vary from those at a non-polluted site; (2) *Vaccinium myrtillus* L. and *Vaccinium vitis-idaea* L. would differ in responses to heavy metal stress; (3) the chosen and examined ecophysiological parameters are good indicators of oxidative stress caused by heavy metals in plants living under field conditions.

## Materials and methods

### Study area

The study was performed in four areas under different levels of anthropogenic stress [immediate vicinity (about 1 km from) of a zinc smelter “Miasteczko Śląskie” (M), iron smelter “ArcelorMittal Poland S.A.” in Dąbrowa Górnicza-Łosień (L) (about 3.5 km from), power plant “Jaworzno III” in Jaworzno (J) (about 1 km from) and nature reserve “Pazurek” in Jaroszowiec Olkuski (P)]. All the sites are situated in southern Poland, in either the Śląskie or Małopolskie provinces. Accurate characterization and maps of the sites are given in a previous works by Kandziora-Ciupa et al. (2013) and Nadgórska-Socha (2016).

Because nature reserve “Pazurek” is situated in close proximity (but not directly) to a number of smelters, in additions to the east (in Poland westerly winds prevail) (Bolesław and Olkusz very strong Zn-Pb mines/smelters – 7 km, many local factories – 3.5 km, and large mine/smelter complex—Upper Silesian Industrial Region – 20 km) we couldn’t considered this study area as completely good control-site, so we decided, in whole manuscript, that this reference site would be referred as “pseudo-control”.

The study was conducted in middle-aged Scots pine forest, growing on sandy acidic soils, mixed with birch (*Betula pendula* L.), European beech (*Fagus sylvatica* L.), and Pedunculate Oak (*Quercus robur* L.), without or with small-scale forest management.

### Sample collection

*Vaccinium myrtillus* L. and *Vaccinium vitis-idaea* L. leaves, stems, and roots and soil samples were collected in mid-June 2012. Each sampling site consisted of 25 × 25 m square in three replicates within which three subsamples bilberry and lingonberry organs and soil samples were randomly collected. The fully matured and undamaged leaves, and the stems and roots of each species were collected from at least 20 different specimens (*V*. *vitis-idaea* L. has not occurred at the site L so organs from this species at this site were not collected). After collection, the samples were covered with plastic bags, deposited in ice, immediately transported to the laboratory, and then frozen until analysis.

The soil samples, after removing surface litter, were taken in the neighborhood of the samples shrubs from a depth of 0–10 cm. At each site soil sub-samples were combined in a composite sample.

### Analysis of metal concentration in the soil and samples of plants

The concentrations of Cd, Pb, Zn, Fe, and Mn were analyzed in individual soil fractions and in the leaves, stems, and roots of *Vaccinium myrtillus* L. and *Vaccinium vitis-idaea* L. The metal content in soil was estimated according to the method of Bouwman et al. 2001 and Ostrowska et al. (2001) in the air-dried soil samples, which were sieved through a 1 mm sieve. Metals were extracted from soil with 0.01 M CaCl_2_ (potentially bioavailable elements) or with 2 M HNO_3_ (acid extracted elements). For the CaCl_2_ extraction, 5 g of soil with 50 ml of 0.01 M CaCl_2_ solution was mechanically shaken for 2 h. The HNO_3_-extractable fraction was obtained by shaking a 10 g soil sample with 100 ml of 2 M HNO_3_ for 1 h. The content of metals was measured in the filtered extracts by inductively coupled plasma-atomic emission spectroscopy (Spectro Analytical Instruments).

For acid extracted elements, single pollution index and Nemerow pollution index were calculated.

Pollution with a given heavy metal (i) was evaluated with the single pollution index (Pi) calculated as the ratio between the metal concentration (Ci) in a soil sample and permitted standard of the same metal (Si):

$${\rm{Pi = }}{{\rm{C}}_{\rm{i}}}/{{\rm{S}}_{\rm{i}}}$$

(Lei et al. 2010; Hu et al. 2013).

Permitted standard for this study was recommended by the Regulation of the Ministry of Environment (2002) about the standards of soil and ground quality (Zn—300 mg kg^−1^; Pb—100 mg kg^−1^; Cd—4 mg kg^−1^).

The overall pollution status of the surface soils by the heavy metals was assessed by the Nemerow pollution index (Pn) (Cheng et al. 2007; Hu et al. 2013):

$${\rm{Pn = }}\sqrt {\left( {{{\rm{P}}_{{\rm{ave}}}}^2{\rm{ + }}{{\rm{P}}_{{\rm{max}}}}^2} \right)} /2$$

where P_ave_ is the average single pollution index of all metals and P_max_ is the maximum value of the single pollution index of all metals. Pollution of the surface soils by the heavy metals was classified into five grades based on the Nemerow pollution index (Pn < 0.7—non-pollution; Pn 0.7–1.0—pollution warning line; Pn 1.0–2.0—low level of pollution; Pn 2.0–3.0—moderate level of pollution; Pn > 3.0—high level of pollution) (Jiang et al. 2014).

In order to determine the heavy metal concentrations in the organs of bilberry and lingonberry, the plant material was washed in a tap with distilled water and dried at 105 °C. A 0.25 g portion of dried plant material was treated with 5 ml of concentrated nitric acid and left for 24 h. Next, the samples were digested at 110 °C until complete digestion was achieved. After digestion, the samples were diluted with deionized water to a volume of 25 ml. Concentration of Cd, Pb, Zn, Fe and Mn were measured using flame absorption spectrometry (Thermo Scientific iCE3500). The quality of the analytical procedure was checked using a reference material (Certified reference Material CTA-OTL-1 Oriental Tobacco Leaves) with the same quantities of samples.

### Metal accumulation efficiency

To evaluate the metal accumulation in plants, translocation factor (TF), mobility ratio (MR), and bioconcentration factor (BCF) were calculated. TF is the ratio of metal concentration in shoots (leaves + stems) and roots. TF > 1 indicates that plants translocates metals effectively from root to shoot (Serbula et al. 2012). MR is the ratio of metal concentration in the shoot (leaves + stems) to its concentration in soil. MR > 1 indicates that the plant is enriched with metals (accumulator), MR1 indicates a rather indifferent behavior of the plant towards metals (indicator), and a MR < 1 shows that the plant excludes metals from uptake (excluder) (Mingorance et al., 2007; Serbula et al. 2012). The bioconcentration factor (BCF) was calculated to measure the ability of each organ (leaves, stems, and roots) to accumulate metals from soil (Hladun et al. 2015). BCF > 1 indicates that particular element is accumulated by leaves, stems, or roots from soil (Yoon et al. 2006; Serbula et al. 2013).

### Analysis of the biochemical parameters of the plants

All ecophysiological parameters were measured in the leaves of *Vaccinium myrtillus* L. and *Vaccinium vitis-idaea* L.

Proline content was determined by the method of Bates et al. (1973). The plant material (0.5 g of) was homogenized in 10 ml of sulfosalicylic acid (3 g per 100 ml) and the homogenate was filtered through Whatman No. 2 filter paper. The reaction mixture containing 2 ml of homogenate, 2 ml acid ninhydrin and 2 ml of glacial acetic acid was incubated at 100 °C for 1 h. The reaction mixture was placed on ice and extracted with 4 ml of toluene. The absorbance was read at 520 nm using toluene as the blank. The proline content was expressed in μmol proline g^−1^ fresh weight.

The content of non-protein thiols was estimated as described by Mass et al. (1987). The plant material was homogenized in a 5 vol/g mixture containing 5-sulphosalicylic acid (2 g per 100 ml) and 1 mM EDTA and sodium ascorbate (0.15 g per 100 ml). The samples were centrifuged at 20,000 *g* for 10 min at 4 °C. Then a 0.5 ml liquid supernatant, 0.5 ml of a 1 M sodium phosphate buffer (pH 8.0) and 100 μl of 10 mM 5,5′-dithio-bis (2-nitrobenzoic acid) (DTNB) were put into test tubes. The absorbance at 415 nm was read 1 min after the addition of DTNB. The number of non-protein SH groups was established based on a curve prepared using L-cysteine and expressed as nmol –SH g^−1^ fresh weight.

To measure the total glutathione concentration (GSHt), plant parts (0.5 g) were homogenized in TCA (trichloroacetic acid, 5 g per 100 ml) and 0.125 mM phosphate buffer (pH 6.3) with 6.3 mM EDTA and were centrifuged at 10,000 *g* for 10 min at 4 °C. Supernatants were used for GSH determination using the DTNB - GSSG reductase recycling procedure according to Anderson (1985). The reaction mixture contained 0.2 ml of supernatant, 0.6 ml of 0.3 mM NADPH, 0.1 mL of 6 mM DTNB and 0.1 ml (0.5 IU ml^−1^) of glutathione reductase. The linear changes in the absorbance of the reaction mixtures were measured at 412 nm and the GSHt was expressed as μmol GSH g^−1^ fresh weight.

Ascorbic acid content was calculated by the formula given by Keller and Schwanger 1977:

$${\rm{Ascorbic acid}}\,\left( {{\rm{mg \times }}{{\rm{g}}^{ - 1}}\,{\rm{f}}{\rm{.w}}{\rm{.}}} \right) = \frac{{\left( {{{\rm{E}}_{\rm{O}}} - {{\rm{E}}_{\rm{S}}} - {{\rm{E}}_{\rm{t}}}} \right)}}{{{\rm{W}} \times {\rm{100}}}} \times 100$$

where V is the volume of the extract, W is the weight of the leaf sample (g), and E_o_, E_s_ and E_t_ are optical densities of a blank sample, a plant sample, and a sample with ascorbic acid, respectively.

The analysis of superoxide dismutase (SOD) was performed in a buffer with 3 mM MgSO_4_, 1 mM dithiotreitol (DTT), 3 mM EDTA (1:5 ratio) and centrifuged at 12,000 *g* for 20 min. The entire procedure was carried out at 4 °C. The reaction was measured spectrophotometrically at 560 nm according to Beauchamp and Fridovich (1971). One unit of SOD was defined as the amount of enzyme activity that was able to inhibit the photoreduction of nitroblue tetrazolium (NBT) to blue formazan by 50%.

For the analysis of guaiacol peroxidase fresh plant material was homogenized in a 100 mM phosphate buffer (pH 6.8). The GPX activity was measured at 470 nm according to Fang and Kao (2000) and Liu et al. (2004) with guaiacol as the substrate. The GPX activity was measured in a reaction mixture (3 ml) containing a 50 mM phosphate buffer (pH 5.8), 1.6 μl H_2_O_2_, 1.5 μl guaiacol and 0.2 ml enzyme extract. The activity was calculated using the extinction coefficient (26 mM^−1^cm^−1^) for tetraguaiacol and was expressed in μmol tetra-guaiacol g ^−1^ fresh weight min^−1^.

### Statistical analysis

The metal content in organs of the studied plants and in the soil and biochemical parameters was analyzed, checked for normality and equality of variance. One-way ANOVA was carried out to compare the differences in soil and plant variables from various sampling sites and significant statistical differences were established using the Tukey’s test, *p* < 0.06 (ANOVA; Statistica 10 package, StatSoft, Inc.). Pearson’s correlation coefficient was calculated for assessing the relationship between estimated metal concentrations and biochemical parameters in the bilberry and lingonberry leaves. CANOCO 4.5 was used to carry out Principal Component Analysis (PCA). Principal Component Analysis assessed the relationships between heavy metal concentrations and biochemical parameters in the leaves of *V. myrtillus* and *V. vitis-idaea*.

## Results

### Heavy metal content and their availability in soil

There were significant differences in the content of the metals studied (HNO_3_ extracted and CaCl_2_ extracted) between the polluted and pseudo-control site (Table 1). Additionally, there was a clear difference in the concentrations of metals between the fraction of soil extracted with HNO_3_ and the fraction extracted with CaCl_2_. Among the metals examined, the highest concentrations of Fe, Pb and Zn were measured in the acid-extracted soil fraction. A several times lower concentration of the metals examined was determined in the fraction of soil extracted with CaCl_2_. The highest level of Cd, Pb and Zn in both fractions was observed at site M (respectively: 40.20, 448.94, 1362.17 mg kg^−1^ after HNO_3_ extraction; 8.95 mg kg^−1^, 18.68, 249.79 after CaCl_2_ extraction) (Table 1). The highest levels of Fe (HNO_3_: 1274.68 mg kg^−1^, CaCl_2_: 14.22 mg kg^−1^) were observed at site P and highest level of Mn we found at sites L and J (L – HNO_3_: 367.48 mg kg^−1^, J – CaCl_2_: 13.07 mg kg^−1^).

**Table 1:** The concentration of selected metals (mg kg^−1^) in fractions of the soils extracted with HNO_3_ and CaCl_2_ (mean values ± SE, *n* = 9). The different letters denote significant differences between the particular metal concentrations (*p* < 0.05)

Site	Cd		Pb		Zn		Fe		Mn		pH
	HNO_3_	CaCl_2_	HNO_3_	CaCl_2_	HNO_3_	CaCl_2_	HNO_3_	CaCl_2_	HNO_3_	CaCl_2_	
M	40.20 ± 5.54b	8.95 ± 1.30b	448.94 ± 26.48b	18.68 ± 5.11b	1362.17 ± 54.84b	249.79 ± 43.82b	1160.72 ± 100.99b	2.13 ± 0.37a	87.61 ± 20.42a	10.19 ± 1.30a	4.5
L	5.86 ± 1.45a	0.46 ± 0.11a	190.88 ± 64.50a	1.01 ± 0.05a	207.98 ± 60.58a	26.78 ± 7.42a	681.39 ± 47.20a	1.11 ± 0.32a	367.48 ± 35.97b	11.14 ± 1.02a	5.1
J	2.87 ± 0.87a	0.51 ± 0.23a	110.17 ± 44.54a	1.37 ± 0.31a	165.32 ± 71.45a	44.19 ± 1.10a	1018.15 ± 33.43b	5.47 ± 2.76a	69.87 ± 14.22a	13.07 ± 0.19a	5.1
P	2.65 ± 0.89a	0.66 ± 0.23a	182.08 ± 56.23a	2.62 ± 0.56a	117.22 ± 35.32a	33.04 ± 11.60a	1274.68 ± 93.35b	14.22 ± 5.30b	70.06 ± 31.35a	8.51 ± 1.39a	4.1

The following descending order of bioavailability was found among the heavy metals: Zn > Cd > Mn > Pb > Fe.

The highest single pollution of all investigated metals were found at site M. Nemerow pollution index ranged from 0.96 at J site to 8.41 in the soil of the most polluted site M (Table 2).

**Table 2:** Single pollution index (SPI) and Nemerow pollution index (NPI) (for acid extracted elements) in soil of investigated sites

Site	SPI-Cd	SPI-Pb	SPI-Zn	NPI
M	10.05	4.49	4.54	8.41
L	1.46	1.91	0.69	1.66
J	0.72	1.10	0.55	0.96
P	0.66	1.82	0.39	1.45

### Metal bioaccumulation in leaves, stems, and roots

Metal bioaccumulation was analyzed in leaves, stems, and roots of *V. myrtillus* and *V. vitis-idaea* (Tables 3 and 4). The highest Cd, Pb, and Zn content in leaves, stems, and roots were found in the plants collected at the M site for both species; additionally at the same site we observed the highest Fe concentrations in all *V. vitis-idaea* organs. The highest bioaccumulation of Mn was noticed in *V. myrtillus* organs.

**Table 3:** The concentrations of heavy metals (mg kg^−1^ d.w.) in the various plant tissue of *V. myrtillus* (mean values ± SE, *n* = 9). The different letters denote significant differences between the particular metal concentrations in the same organ (*p* < 0.05)

Site	Plant organs	Cd	Pb	Zn	Fe	Mn
M	Leaves	7.68 ± 2.31b	222.02 ± 61.58b	308.70 ± 63.44b	121.41 ± 11.44a	133.15 ± 12.28a
	Stems	15.34 ± 2.83b	217.02 ± 42.38b	640.08 ± 20.05b	92.27 ± 17.74a	132.69 ± 28.19a
	Roots	22.91 ± 1.58b	837.47 ± 33.24b	886.75 ± 2.39c	148.47 ± 12.29ab	154.03 ± 2.93a
L	leaves	0.75 ± 0.39a	34.07 ± 13.23a	65.23 ± 15.55a	177.06 ± 4.15a	661.56 ± 10.79c
	Stems	0.97 ± 0.06a	39.99 ± 12.76a	299.80 ± 13.43a	135.89 ± 35.29a	418.28 ± 15.93b
	Roots	1.95 ± 0.07a	45.78 ± 18.06a	149.42 ± 7.53a	195.84 ± 32.05bc	415.35 ± 17.87bc
J	Leaves	0.22 ± 0.09a	20.93 ± 8.34a	57.93 ± 17.81a	164.62 ± 47.23a	873.11 ± 15.55d
	Stems	1.02 ± 0.11a	51.36 ± 37.25a	177.05 ± 14.28a	100.29 ± 15.73a	605.16 ± 76.19b
	Roots	1.10 ± 0.07a	33.86 ± 4.42a	139.42 ± 7.55a	214.27 ± 12.52c	411.16 ± 14.29b
P	Leaves	0.32 ± 0.15a	201.02 ± 98.97b	70.27 ± 1.43a	120.74 ± 32.17a	555.92 ± 56.91b
	Stems	0.24 ± 0.05a	27.23 ± 4.00a	191.40 ± 12.28a	116.69 ± 15.37a	583.90 ± 70.83b
	Roots	1.71 ± 0.04a	98.99 ± 38.21a	208.63 ± 3.40b	139.34 ± 5.84a	450.81 ± 15.95c

**Table 4:** The concentrations of heavy metals (mg kg^−1^ d.w.) in the various plant tissue of *V. vitis-idaea* (mean values ± SE, *n* = 9). The different letters denote significant differences between the particular metal concentrations in the same organ (*p* < 0.05)

Site	Plant organs	Cd	Pb	Zn	Fe	Mn
M	Leaves	14.02 ± 2.96b	461.79 ± 49.04b	427.25 ± 36.32b	178.79 ± 2.31b	79.94 ± 2.10a
	Stems	54.47 ± 0.78b	1381.43 ± 133.38b	1067.95 ± 97.44b	412.02 ± 67.84b	56.87 ± 2.36a
	Roots	55.69 ± 9.28b	1041.58 ± 141.05b	2068.56 ± 253.78b	406.54 ± 70.25c	151.64 ± 14.33a
J	Leaves	0.19 ± 0.03a	27.44 ± 12.94a	62.91 ± 1.61a	112.23 ± 7.73a	126.40 ± 2.11b
	Stems	0.41 ± 0.01a	111.63 ± 2.96a	96.46 ± 4.19a	283.10 ± 2.11a	92.37 ± 3.76b
	Roots	1.23 ± 0.05a	90.84 ± 31.23a	116.18 ± 1.55a	270.14 ± 13.19b	106.29 ± 3.24a
P	Leaves	0.23 ± 0.08a	91.94 ± 36.15a	107.20 ± 5.14a	159.84 ± 5.60c	507.31 ± 0.08c
	Stems	0.27 ± 0.05a	100.93 ± 1.49a	140.56 ± 28.43a	214.08 ± 3.84a	386.12 ± 16.94c
	Roots	2.38 ± 0.78a	87.79 ± 3.46a	178.29 ± 43.39a	140.21 ± 40.16a	338.05 ± 74.68b

### Metal accumulation efficiency

Mean values of translocation factor, mobility ratio, and bioconcentration factors in bilberry and lingonberry at all sampling sites are given in Tables 5 and 6. Effective translocation (TF > 1) in *V. myrtillus* was observed for Pb at J and P sites, for Zn at L site and for Mn at L, J and P sites; in *V. vitis-idaea* was observed for Pb, Zn, and Mn at P site, and additionally for Mn at J site. The values of mobility ratio for Cd, Pb, Zn, and Fe in shoots of both species from contaminated areas showed that their absorption from the soil was not considerable (except Pb at M site for lingonberry). However, we observed very high MR for Mn at all investigated sites for *V. myrtillus* and at J and P site for *V. vitis-idaea*. Also for Mn BCF was greater than one for all organs at all sites for *V. myrtillus* and at sites J and P for *V. vitis-idaea*. BCF also greater than one was observed for Pb in all lingonberry organs at most contaminated site (M) and for this same species for Zn in roots from M and P sites. For *V. myrtillus* BCF > 1 for Pb and Zn in roots was found at M and P sites, respectively, and for Zn in stems from L, J, and P sites.

**Table 5:** Translocation factor (TF), Mobility ratio (MR) and Bioconcentration factors (BCF) (in various plant organs) calculated for *V. myrtillus* (*n* = 9)

			M	L	J	P
Cd	TF		0.51	0.44	0.57	0.17
	MR		0.29	0.15	0.22	0.11
		Leaves	0.19	0.13	0.08	0.12
	BCF	Stems	0.38	0.17	0.36	0.09
		Roots	0.57	0.33	0.09	0.65
Pb	TF		0.27	0.98	1.03	1.13
	MR		0.49	0.19	0.33	0.63
		Leaves	0.49	0.18	0.19	1.10
	BCF	Stems	0.48	0.21	0.47	0.15
		Roots	1.87	0.24	0.31	0.54
Zn	TF		0.53	1.21	0.85	0.63
	MR		0.35	0.88	0.71	1.12
		Leaves	0.23	0.31	0.35	0.60
	BCF	Stems	0.47	1.44	1.07	1.63
		Roots	0.65	0.72	0.84	1.78
Fe	TF		0.72	0.80	0.62	0.85
	MR		0.09	0.23	0.13	0.09
		Leaves	0.10	0.26	0.16	0.09
	BCF	Stems	0.08	0.20	0.10	0.09
		Roots	0.13	0.29	0.21	0.11
Mn	TF		0.86	1.30	1.80	1.27
	MR		1.52	1.47	10.58	8.13
		Leaves	1.52	1.80	12.50	7.93
	BCF	Stems	1.51	1.14	8.66	8.33
		Roots	1.76	1.13	5.88	6.43

**Table 6:** Translocation factor (TF), Mobility ratio (MR) and Bioconcentration factors (BCF) (in various plant organs) calculated for *V. vitis-idaea* (*n* = 9)

			M	J	P
Cd	TF		0.63	0.24	0.11
	MR		0.85	0.10	0.09
		Leaves	0.35	0.07	0.09
	BCF	Stems	1.36	0.14	0.10
		Roots	1.39	0.43	0.90
Pb	TF		0.89	0.83	1.09
	MR		2.05	0.63	0.53
		Leaves	1.03	0.25	0.50
	BCF	Stems	3.08	1.01	0.55
		Roots	2.32	0.82	0.48
Zn	TF		0.37	0.69	0.72
	MR		0.55	0.48	1.05
		Leaves	0.31	0.38	0.91
	BCF	Stems	0.78	0.58	1.19
		Roots	1.52	0.70	1.52
Fe	TF		0.73	0.73	1.41
	MR		0.25	0.19	0.15
		Leaves	0.15	0.11	0.13
	BCF	Stems	0.35	0.28	0.17
		Roots	0.35	0.27	0.11
Mn	TF		0.45	1.03	1.36
	MR		0.78	1.57	6.38
		Leaves	0.91	1.81	7.24
	BCF	Stems	0.65	1.32	5.51
		Roots	1.73	1.52	4.83

### The biochemical status of the plants

**Fig. 1:**
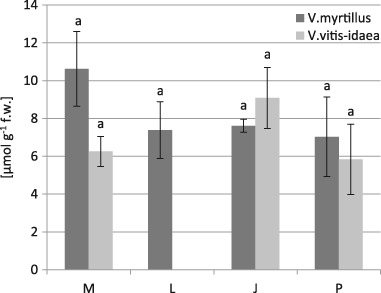
Proline contents (µmol g^−1^ fresh weight) in *V. myrtillus* and *V. vitis-idaea* leaves (mean values ± SE, *n* = 9). *Different letters* above the columns indicate significant differences for the species (*p* < 0.05)

**Fig. 2:**
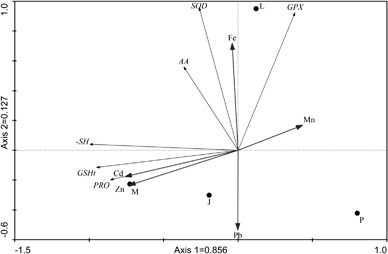
Principal Component Analysis (PCA) biplot of sampling sites and heavy metal concentrations and biochemical parameters in the leaves of *V. myrtillus*

**Fig. 3:**
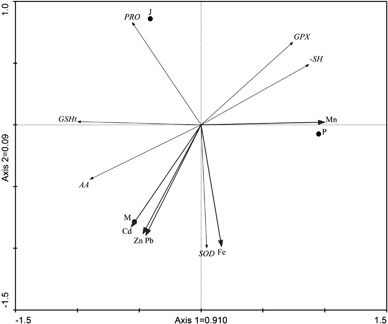
Principal Component Analysis (PCA) biplot of sampling sites and heavy metal concentrations and biochemical parameters in the leaves of *V. vitis-idaea*

There were no significant differences in the proline content between the contaminated and pseudo-control sites and between two investigated species (Fig. 1). We found significant correlations between the proline content and the concentrations of the Cd, Zn, and Mn in the leaves of *V. myrtillus* (Table 7, Fig. 2) and between proline content and the concentration of the Fe in the leaves of *V. vitis-idaea* (Table 8, Fig. 3).

**Table 7:** The correlation coefficients between metal concentration and antioxidant measurements in the leaves of *V*. *myrtillus* (*p* < 0.05); NS-not significant

	Cd	Pb	Zn	Fe	Mn
PRO	0.79	NS	0.77	NS	−0.63
GSHt	NS	NS	0.58	NS	NS
–SH	0.91	NS	0.91	NS	NS
AA	NS	NS	NS	NS	−0.63
SOD	NS	NS	NS	NS	NS
GPX	NS	NS	NS	NS	NS

**Table 8:** The correlation coefficients between metal concentration and antioxidant measurements in the leaves of *V. vitis-idaea* (*p* < 0.05); NS-not significant

	Cd	Pb	Zn	Fe	Mn
PRO	NS	NS	NS	−0.76	NS
GSHt	NS	NS	NS	NS	−0.95
–SH	NS	NS	NS	NS	NS
AA	0.80	0.70	0.75	NS	−0.78
SOD	0.77	0.85	0.84	0.91	NS
GPX	−0.71	−0.67	−0.70	NS	NS

**Fig. 4:**
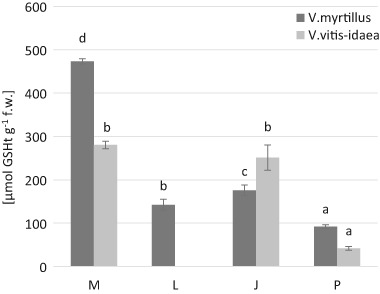
Total glutathione contents (µmol GSHt g^−1^ fresh weight) in *V. myrtillus* and *V. vitis-idaea* leaves (mean values ± SE, *n* = 9). *Different letters* above the columns indicate significant differences for the species (*p* < 0.05)

The greatest concentration of non-protein –SH groups was found in the leaves of *V. myrtillus* at the most contaminated M site (47.78 nmol –SH g^−1^ fresh weight) (Fig. 4). This dependence was confirmed by a positive correlation between non-protein –SH groups and the content of Cd and Zn in the leaves of bilberry (Table 7). At the other sites and in *V. vitis-idaea* leaves the content of NPTs was definitely lower (Fig. 4).

**Fig. 5:**
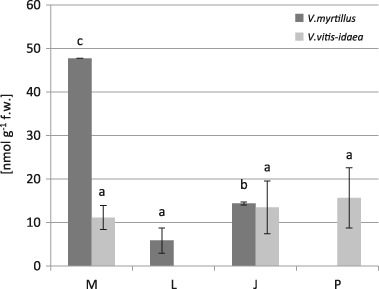
Non-protein –SH groups contents (nmol –SH g^−1^ fresh weight) in *V. myrtillus* and *V. vitis-idaea* leaves (mean values ± SE, *n* = 9). *Different letters* above the columns indicate significant differences for the species (*p* < 0.05)

**Fig. 6:**
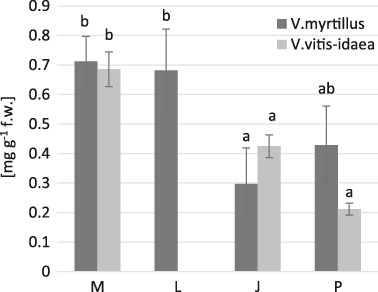
Ascorbic acid contents (mg g^−1^ fresh weight) in *V. myrtillus* and *V. vitis-idaea* leaves (mean values ± SE, *n* = 9). *Different letters* above the columns indicate significant differences for the species (*p* < 0.05)

In the leaves of both investigated species the highest content of glutathione (GSHt) (for *V. myrtillus*: 473.02 μmol GSH g^−1^ f.w.; for *V. vitis-idaea* 280.46 μmol GSH g^−1^) and ascorbic acid (AA) (for *V. myrtillus*: 0.71 mg g^−1^ f.w; for *V. vitis-idaea* 0.68 mg g^−1^ f.w) was detected in the most contaminated area (M) (Fig. 5, Fig. 6). A positive correlation was found between GSHt and Zn in leaves of *V. myrtillus* and between AA and Cd, Pb, Zn in leaves of *V. myrtillus* (Tables 7 and 8; Figs. 2 and 3). Additionally we found a strong negative correlation between Mn and GSHt and AA in *V. vitis-idaea* leaves, and also between Mn and AA in *V. myrtillus* leaves (Tables 7 and 8; Figs. 2 and 3).

**Fig. 7:**
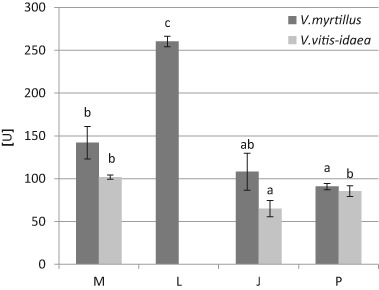
Superoxide dismutase activity (mg g^−1^ fresh weight) in *V. myrtillus* and *V. vitis-idaea* leaves (mean values ± SE, *n* = 9). *Different letters* above the columns indicate significant differences for the species (*p* < 0.05)

An elevated SOD activity in the leaves of *V. myrtillus* and *V. vitis-idaea* in our study was recorded at M and L sites—the highest activity was observed in *V. myrtillus* at L site (260.21 U) (Fig. 7). We found positive correlations between Cd, Pb, Zn, Fe and SOD activity in *V. vitis-idaea* leaves (Table 8, Fig. 3).

**Fig. 8:**
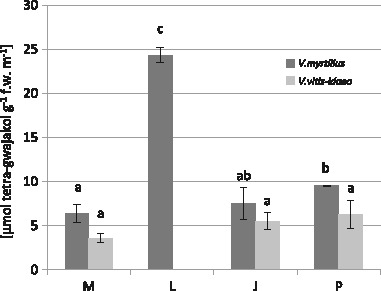
Guaiacol peroxidase activity (µmol tetra-guaiacol g−^1^ fresh weight min^−1^) in *V. myrtillus* and *V. vitis-idaea* leaves (mean values ± SE, *n* = 9). *Different letters* above the columns indicate significant differences for the species (*p* < 0.05)

Guaiacol peroxidase activity was by far the highest in the leaves of *V. myrtillus* from the L site (24.34 µmol tetra-guaiacol g^−1 ^fresh weight min^−1^) (Fig. 8). The lowest GPX activity was observed at M in leaves of both investigated species (for *V. myrtillus*: 6.39 34 µmol tetra-guaiacol g^−1^ fresh weight min^−1^; for *V. vitis-idaea* 3.57 34 µmol tetra-guaiacol g^−1 ^fresh weight min^−1^) (Fig. 8). Guaiacol peroxidase activity in the leaves of lingonberry was negatively correlated with Cd, Pb, and Zn content (Table 8 and Fig. 3).

In *V. myrtillus*, the first two axes of PCA explained 98.3% physiological characteristic variability (85.6% by axis 1; 12,7% by axis 2) (Fig. 2) Similarly, in *V. vitis-idaea* the first two axis of PCA explained 100% of physiological characteristic variability (91% by axis 1 and 9% by axis 2) (Fig. 3).

## Discussion

### Heavy metal content and their availability in soil

Forest ecosystems are known for their high capacity to filter contaminants, including heavy metals (Jamnická et al. 2013). In this study we aimed at evaluating heavy metal accumulation and ecophysiological parameters in *V. myrtillus* and *V. vitis-idaea* growing naturally at contaminated and pseudo-control sites.

Many studies emphasize that the total concentration of heavy metals in soils are useful indicators of the extent of soil contamination but provides a poor indication of plant phytoavailability (Tokalioglu et al. 2000; Wang et al. 2004; Feng et al. 2005; Ortiz and Alcañiz 2006; Xiao et al. 2011; Dao et al. 2012; Boussen et al. 2013). A large part of biomonitoring research consists in the comparison of bioavailable fractions of metals (extracted with CaCl_2_) with their total concentration or pseudo-total (extracted, e.g. 2 M HNO_3_) in soil (Ullrich et al. 1999; Peijnenburg and Jager 2003; Pueyo et al. 2004; Keller and Hammer 2004; Kandziora-Ciupa et al. 2013). Neutral salt extraction (e.g. with CaCl_2_) provides the most useful indication of metal phytoavailability and is more effective for estimating plant availability (Lebourg et al. 1996; Boussen et al. 2013). In our study, higher concentrations of (acid- and CaCl_2_-extracted) Cd, Pb, and Zn were found in the Miasteczko Śląskie area (M) in comparison to other investigated sites. These concentrations (acid extracted) were above permissible levels (Pb—100 mg kg^−1^; Cd—4 mg kg^−1^ Zn—300 mg kg^−1^) according to the Regulations by the Minister of the Environment (2002). Exceeded concentrations were also found for Cd at L site and for Pb at all investigated sites. These results are similar to our previously studies carried out at the same investigated areas (Kandziora-Ciupa et al. 2013; Nadgórska-Socha et al. 2016). Also the single pollution index and Nemerow pollution indices showed serious pollution with heavy metals at M site. The levels of heavy metals at the most polluted site were lower than those found in heap soils left by the historical Zn-Pb ore mining (Stefanowicz et al. 2016).

### Metal bioaccumulation in leaves, stems, and roots

The vascular plants of the forest floor may be good bioindicators to assess the degree of natural environmental pollution, and research on their response to pollution could contribute to a better understanding of the mechanism and factors affecting element bioavailability and plant uptake (Wittig 1992; Jamnická et al. 2013). In this study we found significantly higher levels of Cd, Pb, and Zn in all organs of investigated species collected at the site located near the zinc smelter (M). The levels of these three metals were much higher than values considered as normal, and also exceeded levels considered toxic for plants (according to Kabata-Pendias and Pendias 2001). Higher bioaccumulation of these three metals was found in the lingonberry (especially in leaves) which is related to the fact that it is an evergreen species, retaining its leaves for many years. The obtained concentrations of Cd, Pb, and Zn were largely similar to those reported in field studies by Nadgórska-Socha et al. (2013a) and Stefanowicz et al. (2016) on other species. Definitely lower Cd, Pb, and Zn contents than in the present study were found in the leaves of bilberries by Białońska et al. (2007).

Average Fe concentration in both plants at all investigated sites oscillated around the reference value according by Markert (1992)—150 mg kg^−1^. These results were similar to those given by Parzych (2014) for bilberry shoots from the Słowiński National Park and by Mróz and Demczuk (2010) in *V. myrtillus* shoots from a copper smelter area. Also Nadgórska-Socha et al. (2016) reported similar Fe contents in *Robinia pseudoacacia* and *Melandrium album* leaves from the same investigated areas.

The concentration of Mn in the shoots and roots of *V. myrtillus* and *V. vitis-idaea* at sites classified as less polluted (J and P) in most cases exceeded values considered as toxic to plants (400–1000 mg kg^−1^ according to Kabata-Pendias and Pendias 2001). The results obtained in this study indicate excessive accumulation of Mn in both species’ shoots, with higher concentrations found in *V. myrtillus*. Mróz and Demczuk (2010) suggested that *V. myrtillus* is an accumulator of Mn and its high concentrations suggest a possibility that bilberry uses Mn for some beneficial purposes. Many other authors also mentioned the large capability of bilberry to accumulate Mn (Reimann et al. 2001; Kozanecka et al. 2002; Salemaa et al. 2004; Boyd 2007; Mróz and Demczuk 2010; Kandziora-Ciupa et al. 2013; Parzych 2014). Reeves and Baker (2000) included *V. myrtillus* as manganese hyperaccumulator.

### Metal accumulation efficiency

In our study, metal accumulation efficiency in plant tissues differed considerably between the elements, plant species, and organs. Cd, Pb, Zn, and Fe were accumulated mainly in the roots of *V. myrtillus* and *V. vitis-idaea*. Both species behave as excluders of Cd, Pb, Zn, and Fe—plants that restrict heavy metal transport from roots to shoots (Stefanowicz et al. 2016). TF, MR, and BCF for Mn in most cases were higher than 1, especially in bilberry, which confirmed its tendency to elevated manganese accumulation.

### The biochemical status of the plants

In our study we also compared the antioxidant response in *V. myrtillus* and *V. vitis-idaea* leaves to verify the influence of heavy metals on the physiology of both species in various contaminated environments. Biomaterials such as leaves of higher plants—the site of major physiological processes—have been used to detect the deposition, accumulation, distribustion of metal pollution and physiological responses (Serbula et al. 2012; Kandziora-Ciupa et al. 2013; Deepalakshmi et al. 2014; Nadgórska-Socha et al. 2014).

Proline plays multifarious roles in plant tissues exposed to abiotic stress such as nutritional reserve for growth, stabilization of protein and membranes, osmoprotection, and free radical scavenging (Zouari et al. 2016). This low molecular compounds accumulating in high concentrations in response to a variety of abiotic stress including heavy metals (Kishor and Sreenivasulu 2014). Induction of proline in plants’ response to heavy metals is to a great extent concentration-dependent, and organ and metal specific (Emamverdian et al. 2015). Therefore, the accumulation of proline in plants under heavy metal pollution has been reviewed by many authors (Tantrey and Agnihotri 2010 in *Cicer arietinum* L.; Kumar et al. 2010 in *Rosa hybrida* L.; Kandziora-Ciupa et al. 2016 in *Pinus sylevstris* L.—field study). Our tests found a positively significant effect of Cd and Zn on the accumulation of proline only in the leaves of *V. myrtillus*. In the leaves of this species the highest proline content was observed the vicinity of the zinc smelter (site M) (Fig. 1). An increase in proline level during environmental contamination was also found in *Philadelphus coronarius* leaves (Kafel et al. 2010—field study) and in the needles of *Pinus sylvestris* (Kandziora-Ciupa et al. 2016—field study). Singh et al. (2014) reported an increase in proline accumulation in *Vigna unguiculata* seedlings in response to increasing Zn concentration. On the other hand, our present study found a decreased proline concentration in relation to increased Fe concentration in *V. vitis-idaea* leaves and increased Mn concentration in *V. myrtillus* leaves. Capability of a specific heavy metal to induce proline accumulation may depend on the concentration and specificity of heavy metals, their toxicity threshold, and plant species employed in the trials (Emamverdian et al. 2015). According to Riscitti et al. (2011), an increase in heavy metals concentration raised the proline content to a specific level, and inhibition of proline accumulation occurs beyond a certain threshold of the metal.

Glutathione (GSH), a nonenzymatic antioxidant, is a low molecular weight thiol implicated in a wide range of metabolic process and constitutes an important plant defense system against environmental stresses, including heavy metals (Hossain et al. 2012). Glutathione protects potentially susceptible cysteine-rich proteins from binding free metal ions and respectively affecting their function. After forming nontoxic complexes with metals, GSH simplifies their sequestration away from sensitive sites in cells (Foyer and Noctor 2005; Herbette et al. 2006; Verbruggen et al. 2009; Józefczak et al. 2012). The changes in GSHt levels are dependent on the metal and the part of plant (Arya et al. 2008; Nadgórska-Socha et al. 2013a; Kandziora-Ciupa et al. 2013, 2016).

Similar to previous research on pine needles (Kandziora-Ciupa et al. 2016—field study) and on bilberry leaves (Kandziora-Ciupa et al. 2013—field study), we found a decline in GSHt due to an increased concentration of Mn in lingonberry leaves (Table 8 and Fig. 3). Also Srivastava and Dubey (2011) reported a decline in the level of GSH in Mn-treated rice seedlings. Additionally, many authors reported that exposure to other heavy metals initially resulted in a severe depletion of glutathione (Cd in pine—Schützendübel et al. 2001; Pb in *Raphanus sativus*—El-Beltagi, Mohamed (2010); Pb in *Vicia faba* and *Phaseolus vulgaris*—Piechalak et al. 2002). The decline in the glutathione content of plants may result from the inhibition of enzymes involved in glutathione synthesis by toxic metal ions. In addition, the depletion of the glutathione pool may also be considered to play some role in the synthesis of phytochelatins and other low molecular weight, thiol-rich compounds, or might be attributed to an increased regeneration of ascorbate from dehydroascorbate (Madhava Rao, Sresty 2000; Yadav 2009; Hossain et al. 2012). Pietrini et al. (2003) suggested that higher heavy metals concentration could overload the defense capacity of glutathione. In our study, we also found a positive correlation between GSHt and Fe content in *V. myrtillus* leaves. The same dependence (induction of GSH by exposure to metals) was demonstrated by many authors (Pietrini et al. 2003 in *Phragmites australis* L.; Nadgórska-Socha et al. 2013a in *C.arenosa* and *P.lanceolata-* field study; Anjum et al. 2016 in maize; Nadgórska-Socha et al. 2016 in *R.pseudoaccacia* and *M.album*- field study).

In addition to glutathione, chelation of heavy metals in plants is achieved by cysteine containing metal-binding ligands, including phytochelatins and metallothioneins (Di Baccio et al. 2005). Non-protein thiols contain a high percentage of cysteine sulfhydryl residues and play an important role in metal detoxification process in plants (Sun et al. 2013). Non-protein compounds rich in –SH groups are involved in metal detoxification and/or metal allocation between different organs of the plant, because their main task is to bind metal ions and form non-toxic complexes with metals which are transported from the cytoplasm into the vacuole (Andrade et al. 2010; Yadav 2009; Kandziora-Ciupa et al. 2016—field study). In our study, the highest NPTs content was noticed in *V. myrtillus* leaves at the most polluted site (M); in addition, the content of non-protein thiols was positively related with concentrations of Cd and Zn in bilberry leaves. These results are similar to our previous studies where we observed an increase in NPTs in the leaves of bilberry (Kandziora-Ciupa et al. 2013—field study) and in the needles of pine (Kandziora-Ciupa et al. 2016—field study). Also Nadgórska-Socha et al. (2011, 2016—field studies) found a positive correlation between NPTs and selected heavy metals in the leaves of *Silene vulgaris* and *Robinia pseudoacacia*. Our results were also similar to Sun et al. (2013) who found a significantly increased concentration of NPTs in cabbage treated with Cd. According to Mishra et al. (2006), an increase in NPTs content indicates an ability to tolerate the cellular metal load.

Ascorbic acid is the most abundant, powerful, and water soluble antioxidant preventing or minimizing the damage caused by ROS in plants (Gill and Tuteja 2010). On one hand, in our study we observed its highest levels (in the leaves of both investigated species) at the most contaminated M site and we found a positive correlation between AA content and Cd, Pb, and Zn levels in lingonberry leaves. On the other hand, we found a decrease in AA levels under Mn excess in *V. myrtillus* and *V. vitis-idaea* leaves. In a study of Rai et al. (2013), the ascorbic acid content was higher in the leaves of plants at an industrial site than at a non-industrial site. The same relationship was found by Meerabai et al. (2012) in *Cajanus cajan* L. leaves. Also Demirevska-Kepova et al. (2004) found a significantly higher AA content at elevated Cu concentration in barley plants, compared to control. Increased levels of ascorbic acid have a positive effect on pollution tolerance, which may be due to the defense mechanism of respective plants (Rai and Panda 2014; Nadgórska-Socha et al. 2016—field study). However, Nadgórska-Socha et al. (2016—field study) observed a negative correlation between ascorbic acid content and Pb and Cd concentrations in the leaves of *Robinia pseudoaccacia*, and between AA and Mn in *Melandrium album* leaves. Similar to our results, Demirevska-Kepova et al. (2004) reported a reduction in ascorbic acid under Mn excess in *Hordeum vulgare*. According to Smirnoff (2000), ascorbic acid is the main source of oxalic acid in some species. Immobilization of excess Mn in oxalate crystals has been suggested as a possible detoxification mechanism (El-Jaoual and Cox 1998; González and Lynch 1999), so an AA decrease under Mn redundancy could be explained by an enhanced synthesis of oxalate at the ascorbic acid (Demirevska-Kepova et al. 2004).

The balance between ROS and the antioxidant system is crucial for survival and adaptation of plants growing in soils with relatively constant levels of heavy metals (Słomka et al. 2008). Research on activity of the antioxidant enzymes is important for a better understanding of antioxidant protection (Nadgórska-Socha et al. 2013a). In this study we also examined an antioxidant defense system formed by SOD and GPX.

SOD is known to catalyze to conversion of O_2˙_^-^ to less toxic H_2_O_2_ and O_2_, and is considered to be the first line of defense against elevated levels of ROS in plants (Zouari et al. 2016). The response of SOD to heavy metal stress varies considerably, depending on plant species, tissue, stage of development, utilized metal, and exposure time (Gratão et al. 2005). In our study, an increase in SOD activity under influence of Cd, Pb, Zn, Fe was observed only in the leaves of *V. vitis-idaea*. Nadgórska-Socha et al. (2013a) also found, in field study, an increase in SOD activity under exposure to Fe in the leaves of *Cardaminopsis arenosa* and *Plantago lanceolata*. Zouari et al. (2016) reported that SOD activity increased significantly in the leaves and roots of Cd-treated young olive trees. Also the results of Verma and Dubey (2003) showed an increase in SOD activity in rice plants under the toxic levels of Pb. According to Sharma et al. (2012), an increased activity of SOD often correlated with increased tolerance of plants against environmental stresses.

Peroxidases are antioxidant enzymes which are significant for plants’ growth and development. Activities of these enzymes are changed under both biotic and abiotic stress conditions, and so are used as a potential indicator of metal toxicity (Radotić et al. 2000; Macfarlane and Burchett 2001; Baycu et al. 2006; Doğanlar and Atmaca 2011; Kandziora-Ciupa et al. 2013). Guaiacol peroxidase is associated with many important biosynthetic processes and defense against abiotic and biotic stresses and is widely accepted as a “stress enzyme” (Erofeeva 2015). Despite the fact that many authors report increased GPX activity in response to elevated heavy metal concentrations (Doğanlar and Atmaca 2011; Kafel et al. 2010; Nadgórska-Socha et al. 2013a; Nadgórska-Socha et al. 2013b—all field studies) in our study, we observed a significant decrease correlated with increased Cd, Pb, and Zn contents, although again only in lingonberry leaves. We found a similar dependence in our previous studies where we reported a decrease in GPX activity under the exposure to Cd, Pb, and Zn in bilberry leaves (Kandziora-Ciupa et al. 2013) and exposure to Zn in spruce needles (Kandziora-Ciupa et al. 2016—field study). Also Nadgórska-Socha et al. (2013a—field study) found a negative correlation between GPX activity and Cd content in *Cardaminopsis arenosa*.

Stress intensity may be linked to an increase or decrease in oxidative metabolism. It is generally accepted that enzymatic activity decreases in higher heavy metal concentrations (Słomka et al. 2008).

## Conclusions

In connection to our hypothesis, we may conclude that much higher Cd, Pb, Zn, and Fe concentrations were found in *V. myrtillus* and *V. vitis-idaea* grown at the most polluted site (located near the zinc smelter) in comparison with cleaner areas, and definitely higher bioaccumulation of these metals was found in lingonberry organs. Increased Mn accumulation was found at less polluted sites (including the outskirts of the Pazurek Nature Reserve) and we noticed the large capability of bilberry to accumulate Mn.

Anti-oxidant response to heavy metal stress differed between *V. myrtillus* and *V. vitis-idaea*. However, we did not find a single general marker of heavy metal contamination in the two investigated species. In bilberry, elevated heavy metal concentrations caused an increase in proline, GSHt, and non-protein –SH groups content. In lingonberry the increased heavy metal accumulation induced an increase in ascorbic acid content, SOD activity, and a decrease in GPX activity. In both species increased Mn accumulation caused a decrease in anti-oxidative response.

The selected ecophysiological parameters may be good biochemical markers of stress caused by heavy metal and we do realize that in natural conditions these parameters are influenced by various factors, which we weren’t studied. Therefore, it is important to continue this type of research on plants growing in natural conditions.

## Abbreviations

SPI: Single pollution index
NPI: Nemerow pollution index
NPTs: Non-protein thiols
GSHt: Glutathione total
AA: Ascorbic acid
SOD: Superoxide dismutase
GPX: Guaiacol peroxidase
TF: Translocation factor
MR: Mobility ratio
BCF: Bioconcentration factor

## References

[CR1] Anderson ME (1985). Determination of glutathione and glutathione disulfide in biological samples. Methods Enzymol.

[CR2] Andrade SAL, Gratão PL, Azevedo RA, Silveira APD, Schiavinato MA, Mazzafera P (2010). Biochemical and physiological changes in Jack bean under mycorrhizal symbiosis growing in soil with increasing Cu concentrations. Environ Exp Bot.

[CR3] Anjum SA, Tanveer M, Hussain S, Bao M, Wang LC, Khan I, Ehsanullah Tung SA, Samad RA, Shahzad B (2015). Cadmium toxicity in Maize (Zea mays L.): consequences on antioxidative systems, reactive oxygen species and cadmium accumulation. Environ Sci Pollut Res.

[CR4] Anjum SA, Tanveer M, Hussain S, Ehsanullah Wang LC, Khan I, Samad RA, Tung SA, Anam M, Shahzad B (2015). Morpho-physiological growth and yield responses of two contrasting maize cultivars to cadmium exposure. Clean Soil Air Water.

[CR5] Anjum SA, Tanveer M, Hussain S (2016). Osmoregulation and antioxidant production in maize under combined cadmium and arsenic stress. Environ Sci Pollut Res.

[CR6] Arya SK, Khalique S, Roy BK (2008). Glutathione and cysteine biosynthesis in two varieties of Abelmoschus esculentus in response to mine spoil. J Environ Biol.

[CR7] Bates L, Waldren R, Teare D (1973). Rapid determination of free proline for water-stress studies. Plant Soil.

[CR8] Baycu G, Tolunay D, Özden H, Guenenebakan S (2006). Ecophysiological and seasonal variations in Cd, Pb, Zn and Ni concentrations in the leaves of urban deciduous trees in Istanbul. Environ Pollut.

[CR9] Beauchamp C, Fridovich I (1971). Superoxide dismutase: improved assays and an assay applicable to acrylamide gels. Anal Biochem.

[CR10] Białońska D, Zobel A, Kuraś M, Tykarska T, Sawicka-Kapusta K (2007). Phenolic compounds and cell structure in bilberry leaves affected by emissions from a Zn–Pb smelter. Water Air Soil Poll.

[CR11] Boussen S, Soubrand M, Bril H, Ouerfelli K, Abdeljaouad S (2013). Transfer of lead, zinc and cadmium from mine tailings to wheat (Triticum aestivum) in carbonated Mediterranean (Northern Tunisia) soils. Geoderma.

[CR12] Bouwman L, Bloem J, Römkens P, Boon G, Vangronsveld J (2001). Beneficial effects of the growth of metal tolerant grass on biological and chemical parameters in copper - and zinc contaminated sandy soils. Minerva Biotech.

[CR13] Boyd RS (2007). The defense hypothesis of elemental hyperaccumulation: status, challenges and new directions. Plant Soil.

[CR14] Cheng J, Shi Z, Zhu Y (2007). Assessment and maping of environmental quality in agricultural soils of Zhejiang Province, China. Huangjin Kexue.

[CR15] Cyjetko P, Tolic S, Sikic S, Balen B, Tkalec M, Vidaković-Cifrek Z, Pavlica M (2010). Effect of copper on the toxicity and genotoxicity of cadmium in duckweed (Lemna minor L.). Arh Hig Rada Toksikol.

[CR16] Dao L, Morrison L, Kiely G, Zhang Ch (2012) Spatial distribution of potentially bioavailable metals in surface soils of a contaminated sports ground in Galway, Ireland. Environ Geochem Health 35(2):227–238 10.1007/s10653-012-9478-7 22864559

[CR17] Das P, Nutan KK, Singla-Pareek SL, Pareek A (2015). Oxidative environment and redox homeostasis in plants: dissecting out significant contribution of major cellular organelles Front. Environ Sci.

[CR18] Deepalakshmi AP, Ramakrishnaiah H, Ramachandra YL, Kumar NN (2014). Leaves of higher plants as indicators of heavy metal pollution along the Urban Roadways. Int J Sci Technol.

[CR19] Demirevska-Kepova K, Simova-Stoilova L, Stoyanova Z, Holzer R, Feller U (2004). Biochemical changes in barley plants after excessive supply of cooper and manganese. Environ Exp Bot.

[CR20] Di Baccio D, Kopriva S, Sebastiani L, Rennenberg H (2005). Does glutathione metabolism have a role in the defence of poplar against zinc excess?. New Phytologist.

[CR21] Doğanlar Z, Atmaca M (2011). Influence of airborne pollution on Cd, Zn, Pb, Cu, and Al accumulation and physiological parameters of plant leaves in Antakya (Turkey). Water Air Soil Poll.

[CR22] El-Beltagi H, Mohamed AA (2010). Changes in nonprotein thiols, some antioxidant enzymes activity and ultrastructural alteration in radish plant (Raphanus sativus L) grown under lead toxicity. Not Bot Hort Agrobot Cluj.

[CR23] El-Jaoual T, Cox DA (1998). Manganese toxicity in plants. J Plant Nutr.

[CR24] Emamverdian A, Ding Y, Mokhberdoran F, Xie Y (2015) Heavy metal stress and some mechanisms of plant defense response. Sci World J. doi:10.1155/2015/756120 10.1155/2015/756120 PMC4321847 25688377

[CR25] Erofeeva EA (2015). Dependence of guaiacol peroxidase activity and lipid peroxidation rate in drooping birch (Betula pendula Roth) and Tillet (Tilia cordata Mill) leaf on motor traffic pollution intensity. Dose-Response.

[CR26] Fang WC, Kao C (2000). Enhanced peroxidase activity in rice leaves in response to excess iron, copper and zinc. Plant Sci.

[CR27] Feng MH, Shan XQ, Zhang SZ, Wen B (2005). Comparison of a rhizosphere-based method with other one-step extraction methods for assessing the bioavailability of soil metals to wheat. Chemosphere.

[CR28] Foyer CH, Noctor G (2005). Oxidant and antioxidant signalling in plants: a re-evaluation of the concept of oxidative stress in a physiological context. Plant Cell Environ.

[CR29] Ganthaler A, Mayr S (2015). Dwarf shrub hydraulics: two vaccinium species (Vaccinium myrtillus, Vaccinium vitis-idaea) of the European Alps compared. Physiol Plant.

[CR30] Gill SS, Tuteja N (2010). Reactive oxygen species and antioxidant machinery in abiotic stress tolerance in crop plants. Plant Physiol Bioch.

[CR31] González A, Lynch J (1999). Subcellular and tissue compartmentation in bean leaves under Mn toxicity stress. Austr J Plant Physiol.

[CR32] Gratão PL, Monteiro CC, Antunes AM, Peres LEP, Azevedo RA (2008). Acquired tolerance of tomato (*Lycopersicon esculentum* cv. Micro-Tom) plants to cadmium-induced stress. Ann Appl Biol.

[CR33] Gratão PL, Polle A, Lea PJ, Azevedo RA (2005). Making the life of heavy metal-stressed plants a little easier. Funct Plant Biol.

[CR34] Herbette S, Taconnat L, Hugouvieux V (2006). Genome-wide transcriptome profiling of the early cadmium response of Arabidopsis roots and shoots. Biochimie.

[CR35] Hladun KR, Parker DR, Trumble JT (2015). Cadmium, copper, and lead accumulation and bioconcentration in the vegetative and reproductive organs of Raphanus sativus: implications for plant performance and pollination. J Chem Ecol.

[CR36] Hossain MA, Piyatida P, Teixeira da Silva JA, Fujita M (2012) Molecular mechanism of heavy metal toxicity and tolerance in plants: central role of glutathione in detoxification of reactive oxygen species and methylglyoxal and in heavy metal chelation. J Bot 2012:1–37

[CR37] Hu Y, Liu X, Bai J, Shih K, Zeng E, Cheng H (2013). Assessing heavy metal pollution in the surface soils of a region that had undergone three decades of intense industrialization and urbanization. Environ Sci Pollut Res.

[CR38] Hu Y, Wang D, Wei L, Zhang X, Song B (2014). Bioaccumulation of heavy metals in plant leaves from Yan’an city of the Loess Plateau, China. Ecotox Environ Safe.

[CR39] Jamnická G, Váľka J, Bublinec E (2013). Heavy metal accumulation and distribution in forest understory herb species of Carpathian beech ecosystems. Chem Spec Bioavailab.

[CR40] Jiang X, Lu WX, Zhao HQ, Yag QC, Yang ZP (2014). Potential ecological risk assessment and prediction of soil heavy metal pollution around coal gangue dump. Nat Hazards Earth Syst Sci.

[CR41] Józefczak M, Remans T, Vangronsveld J, Cuypers A (2012). Glutathione is a key player in metal-induced oxidative stress defenses. Int J Mol Sci.

[CR42] Kabata-Pendias A, Pendias H (2001). Trace elements in soils and plants.

[CR43] Kafel A, Nadgórska-Socha A, Gospodarek J, Babczyńska A, Skowronek M, Kandziora M, Rozpendek K (2010). The effects of Aphis fabae infestation on the antioxidant response and heavy metal content in field grown Philadelphus coronarius plants. Sci Total Environ.

[CR44] Kandziora-Ciupa M, Ciepał R, Nadgórksa-Socha A, Barczyk G (2013). A comparative study of heavy metal accumulation and antioxidant responses in *Vaccinium myrtillus* L. leaves in polluted and non-polluted areas. Environ Sci Pollut Res.

[CR45] Kandziora-Ciupa M, Ciepał R, Nadgórska-Socha A, Barczyk G (2016). Accumulation of heavy metals and antioxidant responses in Pinus sylvestris L. needles in polluted and non-polluted sites. Ecotoxicology.

[CR46] Keller C, Hammer D (2004). Metal availability and soil toxicity after repeated croppings of Thlaspi caerulescens in metal contaminated soils. Environ Pollut.

[CR47] Keller T, Schwanger H (1977). Air pollution and ascorbic acid. Eur J Forest Pathol.

[CR48] Kishor PBK, Sreenivasulu N (2014). Is proline accumulation per se correlated with stress tolerance or is proline homeostasis a more critical issue?. Plant Cell Environ.

[CR49] Kolari P, Pumpanen J, Kulmala L, Ilvesniemi H, Nikinmaa E, Grönholm T, Hari P (2006). Forest floor vegetation plays an important role in photosynthetic production of boreal forests. For Ecol Manage.

[CR50] Kozanecka T, Chojnicki J, Kwasowski (2002). Content of heavy metals in plant from pollution-free regions. Polish J Environ Stud.

[CR51] Kumar N, Pal M, Singh A, Kumar SaiRam R, Srivastava GH (2010). Exogenous proline alleviates oxidative stress vase life in rose (*Rosa hybrid*a L.‘Grand Gala‘). Sci Hortic.

[CR52] Lebourg A, Sterckeman T, Ciesielski H, Proix N (1996). Intérêt de différents réactifs d’extraction chimique pour l'évaluation de la biodisponibilité des métaux en traces des sols. Agronomie.

[CR53] Lei M, Zhang Y, Khan S, Qin P, Liao B (2010). Pollution, fractionation and mobility of Pb, Cd, Cu and Zn in garden and paddy soils from Pb/Zn mining area. Environ Monit Assess.

[CR54] Liu J, Xiong Z, Li T, Huang H (2004). Bioaccumulation and ecophysiological responses to copper stress in two populations of *Rumex dentatus* L. from Cu contaminated and non-contaminated sites. Environ Exp Bot.

[CR55] Liu Y, Liu S, Liu D, Wei Y, Liu C, Yang Y, Tao C, Liu W (2014). Exploiting EST databases for the development and characterization of EST-SSR markers in blueberry (Vaccinium) and their cross-species transferability in Vaccinium spp. Sci Hortic (Amsterdam).

[CR56] Macfarlane GR, Burchett MD (2001). Photosynthetic pigments and peroxidase activity as indicators of heavy metal stress in the grey mangrove *Avicennia marina* (Forsk.) Veirh. Mar Pollut Bull.

[CR57] Madhava Rao KV, Sresty TVS (2000). Antioxidative parameters in the seedling of pigeonpea (*Cajanus cajan* L. Millspaugh) in response to Zn and Ni stresses. Plant Sci.

[CR58] Markert B (1992). Presence and significance of naturally occurring chemical elements of the periodic system in the plant organism. Vegetatio.

[CR59] Mass F, De Kok L, Peters J, Kuiper PA (1987). Comparative study on the effects of H_2_S and SO_2_ fumigation on the growth and accumulation of sulfate and sulfhydryl compounds in *Trifolium pratense* L., *Glycine max* Merr., *Phaseolus vulgaris* L. J Exp Bot.

[CR60] Meerabai G, Venkata RC, Rasheed M (2012). Effect of industrial pollution on physiology of Cajanus cajan (L.) – Fabaceae. Intl J Environ Sci.

[CR61] Mingorance MD, Valdés B, Rossini Oliva S (2007). Strategies of heavy metal uptake by plants growing under industrial emissions. Environ Int.

[CR62] Mishra S, Srivastava S, Tripathi RD, Kumar R, Seth CS, Gupta DK (2006). Lead detoxification by coontail (Ceratophyllum demersum L.) involves induction of phytochelatins and antioxidant system in response to its accumulation. Chemosphere.

[CR63] Mróz L, Demczuk M (2010). Contents of phenolics and chemical elements in bilberry (Vaccinium myrtillus L.) leaves from copper smelter area. Pol J Ecol.

[CR64] Nadgórska-Socha A, Kafel A, Kandziora-Ciupa M, Gospodarek J, Zawisza-Raszka A (2013). Accumulation of heavy metals and antioxidant responses in Vicia faba plants grown on monometallic contaminated soil. Environ Sci Pollut Res.

[CR65] Nadgórska-Socha A, Kandziora-Ciupa M, Ciepał R (2011). Effects of Zn, Cd, Pb on physiological reponse of Silene vulgaris plants from selected populations. Polish J Environ Stud.

[CR111] Nadgórska-Socha A, Kandziora-Ciupa M, Ciepał R (2015) Element accumulation, distribution, and phytoremediation potential in selected metallophytes growing in a contaminated area. Environ Monit Assess 187(7):441. doi:10.1007/s10661-015-4680-6 10.1007/s10661-015-4680-6 26088758

[CR66] Nadgórska-Socha A, Kandziora-Ciupa M, Ciepał R, Barczyk G (2016). Robinia pseudoacacia and Melandrium album in trace elements biomonitoring and air pollution tolerance index study. J Environ Sci Technol.

[CR67] Nadgórska-Socha A, Ptasiński B, Kita A (2013). Heavy metal bioaccumulation and antioxidative responses in Cardaminopsis arenosa and Plantago lanceolata leaves from metalliferous and non-metalliferous sites: a field study. Ecotoxicology.

[CR68] Nilsson MC, Wardle WA (2005). Understory vegetation as a forest ecosystem driver: evidence from the northern Swedish boreal forest. Front Ecol Environ.

[CR69] Ortiz O, Alcañiz JM (2006). Bioaccumulation of heavy metals in Dactylis glomerata L. growing in a calcareous soil amended with sewage sludge. Bioresour Technol.

[CR70] Ostrowska A, Gawliński S, Szczubiałka Z (2001) In: Method of analysis and estimate soil and plants property, Catalogue of the Environmental Prootection Institute Warsaw pp 334–336 (in Polish)

[CR71] Parzych A (2014). The heavy metal content of soil and shoots of Vaccinium myrtillus L. in the Słowiński National Park. Forest Res Papers.

[CR72] Peijnenburg WJGM, Jager T (2003). Monitoring approaches to assess bioaccessibility and bioavailability of metals: matrix issues. Ecotoxicol Environ Saf.

[CR73] Piechalak A, Tomaszewska B, Baralkiewicz D, Małecka A (2002). Accumulation and detoxification of lead ions in legumes. Phytochemistry.

[CR74] Pietrini F, Iannelli MA, Pasqualini S, Massacci A (2003). Interaction of cadmium with glutathione and photosynthesis in developing leaves and chloroplasts of Phragmites australis (Cav.) Trin. Ex Steudel. Plant Physiol.

[CR75] Polatschek A (1999). Flora von Nordtirol, Osttirol und Vorarlberg, Band 2.

[CR76] Pueyo M, Lopez-Sanchez JF, Rauret G (2004). Assessment of CaCl2, NaNO3 and NH4NO3 extraction procedures for the study of Cd, Cu, Pb and Zn extractability in contaminated soils. Anal Chimica Acta.

[CR77] Radotić K, Ducić T, Mutavdžic D (2000). Changes in peroxidase activity and isoenzymes in spruce needles after exposure to different concentrations of cadmium. Environ Exp Bot.

[CR78] Rai P, Panda KLSS, Chutia BM, Singh MM (2013). Comparative assessment of air pollution tolerance index (APTI) in the industrial (Rourkela) and non-industrial area (Aizawl) of India: an eco management approach. Afr J Environ Sci Tech.

[CR79] Rai PK, Panda LS (2014). Dust capturing potential and air pollution tolerance index (APTI) of some roadside tree vegetation in Aizawl, Mizoram, India: an Indo-Burma hot spot region. Air Quality Atmosphere Health.

[CR80] Reeves RD, Baker AJM, Raskin I, Ensley BD (2000). Metal-accumulating plants. Phytoremediation of toxic metals: using plants to clean up the environment.

[CR81] Regulation by the Minister of Environment dated 9 September (2002) Official Gazette No. 165, Pos. 1359th (in Polish)

[CR82] Reimann C, Koller F, Frengstad B, Kashulina G, Niskavaara H, Englmaier P (2001). Comparison of the element composition in several plant species and their substrate from a 1500000-km2 area in Northern Europe. Sci Total Environ.

[CR83] Rodriguez A, Kouki J (2015). Emulating natural disturbance in forest management enhances pollination services for dominant Vaccinium shrubs in boreal pine dominated forests. Forest Ecology Manage.

[CR84] Salemaa M, Derome J, Helmisaari H-S, Nieminen T, Vanha-Majamaa I (2004). Element accumulation in boreal bryophytes, lichens and vascular plants exposed to heavy metal and sulfur deposition in Finland. Sci Total Environ.

[CR85] Schützendübel A, Schwanz P, Teichmann T, Gross K, Langenfeld-Heyser R, Godbold DL, Polle A (2001). Cadmium-induced changes in antioxidative systems, hydrogen peroxide content and differentiation in Scots Pine roots. Plant Physiol.

[CR86] Sebald O, Seybold S, Philippi G (1993). Die Farn‐ und Blütenpflanzen Baden‐Württembergs, Band 2.

[CR87] Serbula SM, Kalinovic TS, Ilic AA, Kalinovic JV, Steharnik MM (2013). Assessment of airborne heavy metal pollution using Pinus spp. and Tilia spp. Aerosol Air Qual Res.

[CR88] Serbula SM, Milljkovic DDJ, Kovacevic RM, Ilic AA (2012). Assessment of airborne heavy metal pollution using plant parts and topsoil. Ecotoxicol Environ Saf.

[CR89] Sharma P, Jha AB, Dubey RS, Pessarakli M (2012) Reactive oxygen species, oxidative damage, and antioxidative defense mechanism in plants under stressful conditions. J Bot 2012:1–26

[CR90] Singh J, Hembram P, Basak J (2014). Potential of Vigna unguiculata as a phytoremediation plant in the remediation of Zn from contaminated soil. Am J Plant Sci.

[CR91] Słomka A, Libik-Konieczny M, Kuta E, Miszalski Z (2008). Metalliferous and non-metalliferous populations of Viola tri-color represent similar mode of antioxidative response. J Plant Physiol.

[CR110] Smirnoff N, Wheeler GL (2000) Ascorbic acid in plants: biosynthesisand function. Crit Rev Plant Sci 19:267–290 10.1080/10409230008984166 11005203

[CR92] Srivastava S, Dubey RS (2011). Manganese-excess induces oxidative stress, lowers the pool of antioxidants and elevates activities of key antioxidative enzymes in rice seedlings. Plant Growth Regul.

[CR93] Stefanowicz AM, Stanek M, Woch MW (2016). High concentrations of heavy metals in beech forest understory plants growing on waste heaps left by Zn-Pb ore mining. J Geochem Explor.

[CR94] Sun J, Cui J, Luo C, Gao L, Chen Y, Shen Z (2013). Contribution of cell walls, nonprotein thiols, and organic acids to cadmium resistance in two cabbage varieties. Arch Environ Contam Toxicol.

[CR95] Tantrey MS, Agnihotri RK (2010). Chlorophyll and proline content of gram (Cicer arietinum L.) under cadmium and mercury treatment. Res J Agric Sci.

[CR96] Taulavuori K, Laine K, Taulavuori E (2013). Experimental studies on *Vaccinium myrtillus* and *Vaccinium vitis-idaea* in relation to air pollution and global change at northern high latitudes: A review. Environ Exp Bot.

[CR98] Tkalec M, Stefanic PP, Cvjetko P, Sikic S, Pavlica M, Balen B (2014). The effects of cadmium-zinc interactions on biochemical responses in tobacco seedlings and adult plants. PLoS ONE.

[CR99] Tokalioglu S, Kartal S, Elc L (2000). Determination of heavy metals and their speciation in lake sediments by flame atomic absorption spectrometry after a four-stage sequential extraction procedure. Anal Chim Acta.

[CR100] Ullrich SM, Ramsey MH, Helios-Rybicka E (1999). Total and exchangeable concentrations of heavy metals in soils near Bytom, an area of Pb/Zn mining and smelting in Upper Silesia, Poland. Appl Geochem.

[CR101] Verbruggen N, Hermans C, Schat H (2009). Mechanisms to cope with arsenic or cadmium excess in plants. Curr Opin Plant Biol.

[CR102] Verma S, Dubey RS (2003). Lead toxicity induces lipid peroxidation and alters the activities of antioxidant enzymes in growing rice plants. Plant Sci.

[CR103] Wang C, Shen Z, Li X, Luo C, Chen Y, Yang H (2004). Heavy metal contamination of agricultural soils and stream sediments near a copper mine in Tongling, People’s Republic of China. Bull Environ Contam Toxicol.

[CR104] Wilde KL, Stauber JL, Markich SJ, Franklin NM, Brown PL (2006). The effect of pH on the uptake and toxicity of copper and zinc in a tropical freshwater alga (Chlorella sp.). Arch Environ Contam Toxicol.

[CR112] Wittig R (1992) Die Eignung der Krautschicht von Wäldern zum Biomonitoring von Schwermetallen. Beih Veröff Natursch Landschaftspfl 64:137–145

[CR106] Xiao R, Bai JH, Wang QG, Gao HF, Huang LB, Liu XH (2011). Assessment of heavy metal contamination of wetland soils from a typical aquatic–terrestrial ecotone in Haihe River Basin, North China. CLEAN-Soil, Air Water.

[CR107] Yadav SK (2009). Heavy metals toxicity in plants: An overview on the role of glutathione and phytochelatins in heavy metal stress tolerance of plants. South Afr J Bot.

[CR108] Yoon J, Cao X, Zhou Q, Ma LQ (2006). Accumulation of Pb, Cu, and Zn in native plants growing on a contaminated Florida site. Sci Total Environ.

[CR109] Zouari M, Ben Ahmed Ch, Zorrig W, Elloumi N, Rabhi M, Delmail D, Ben Rouina B, Labrousse P, Ben Abdallah F (2016). Exogenous proline mediates alleviation of cadmium stress by promoting photosynthetic activity, water status and antioxidative enzymes activities of young date palm (Phoenix dactylifera L.). Ecotoxicol Environ Saf.

